# Cerebral Septic Embolism From Infective Endocarditis: Microbiological Confirmation From Mechanical Thrombectomy Specimen

**DOI:** 10.7759/cureus.91759

**Published:** 2025-09-07

**Authors:** Sujith Jayaprakash, Jithin Bose, Gigy V Kuruttukulam

**Affiliations:** 1 Medicine, Government Medical College, Thiruvananthapuram, Thiruvananthapuram, IND; 2 Neurology, Rajagiri Hospital, Aluva, IND

**Keywords:** enterococcus faecalis, infective endocarditis, mechanical thrombectomy (mt), sceptic embolism, thrombus microbiology

## Abstract

Neurological complications occurring in patients with infective endocarditis (IE) are a major determinant of morbidity and mortality. Among these, ischemic stroke secondary to septic embolism is the most common and often results from embolization of friable vegetations into the cerebral circulation. Such events typically occur early in the disease course, frequently within the first two weeks of antimicrobial therapy. The embolic material not only causes vascular occlusion but may also introduce infection into cerebral tissue, increasing the risk of secondary abscess formation, meningitis, and mycotic aneurysms. Management of acute large-vessel occlusion (LVO) stroke in IE poses unique challenges; standard intravenous thrombolysis carries a heightened risk of intracranial hemorrhage, while endovascular therapy must account for friable clot morphology and possible vessel fragility from infection-related vasculitis. The following case demonstrates the diagnosis and successful mechanical thrombectomy of an LVO in a postpartum woman with IE, with microbiological confirmation of *Enterococcus faecalis *directly from the retrieved thrombus, which was initially isolated from blood culture. *E. faecalis* is common in the genitourinary flora. This case highlights the importance of early diagnosis, vigilant monitoring for embolic events, and timely surgical intervention in infective endocarditis.

## Introduction

Infective endocarditis (IE) is a serious and often fatal condition with an incidence of 3-10 cases per 100,000 population and in-hospital mortality of 18-25% [1]. Embolic complications are frequent, occurring in 20-50% of cases, with ischemic stroke reported in up to 35% [2]. The risk is greatest at diagnosis and during the first two weeks of antimicrobial therapy and is strongly influenced by vegetation size, mobility, mitral valve involvement, and causative organisms such as *Staphylococcus aureus* and *Enterococcus faecalis* [3].

The postpartum period is a transient hypercoagulable state, which increases susceptibility to thromboembolic complications. Although IE is rare in this setting, when it does occur, the risk of systemic and cerebral embolism is amplified. *E. faecalis* accounts for about 10% of IE overall but less than 2% of peripartum cases, yet it is clinically significant due to its resistance profile and high complication rates [4]. Clinical manifestations typically include fever, new or changing murmurs, and peripheral signs such as Janeway lesions and Osler’s nodes. In postpartum women presenting with stroke, differential diagnoses include venous thromboembolism, cerebral venous sinus thrombosis, postpartum angiopathy, and cardioembolic stroke [5].

Diagnosis of IE is based on the modified Duke criteria, with blood cultures and echocardiography as the cornerstones. Rarely, as in the present case, causative organisms may be identified in both blood cultures and embolic material retrieved during thrombectomy, providing direct microbiological confirmation [6]. Treatment requires prolonged intravenous antibiotics tailored to sensitivity. For *E. faecalis*, synergistic regimens such as ampicillin plus ceftriaxone or gentamicin are standard. Early surgery is recommended in cases with large vegetations, recurrent embolism, heart failure, or persistent infection, with evidence showing that early intervention reduces mortality and embolic recurrence [7].

## Case presentation

**Figure 1:**
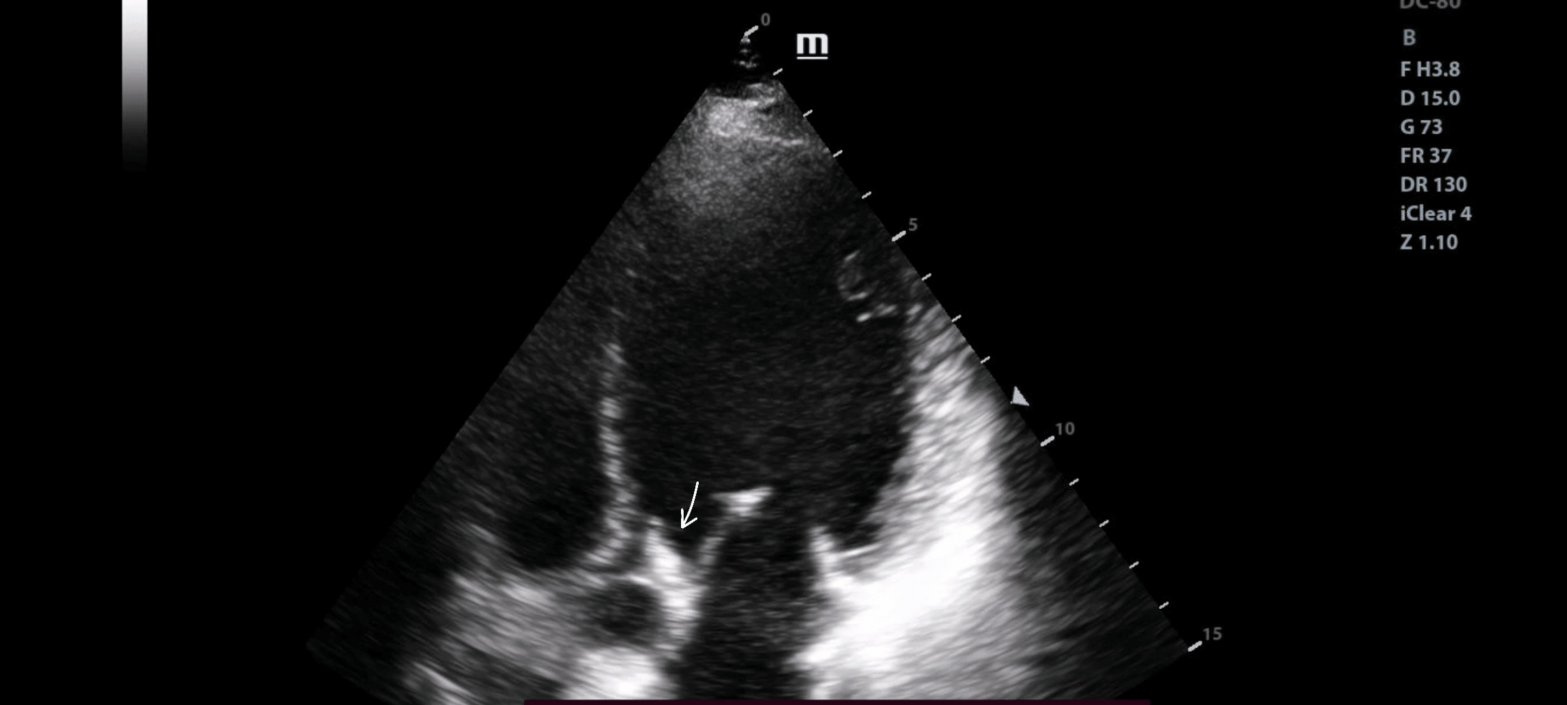
Echocardiogram on the day of admission. Normal chamber dimensions, no significant regional wall motion abnormality, adequate left ventricular systolic function, vegetation measuring 1.3 × 0.6 cm attached to the aortic valve, causing severe aortic regurgitation and no aortic stenosis, Grade 2 mitral regurgitation, mild tricuspid regurgitation, right ventricular systolic pressure of 26 mmHg plus right atrial pressure, no pulmonary artery hypotension. Good right ventricular function, patent foramen ovale present, intact interventricular septum, no pericardial effusion, inferior vena cava 1.5 cm collapsing with respiration, and ejection fraction of 50%.

**Figure 2:**
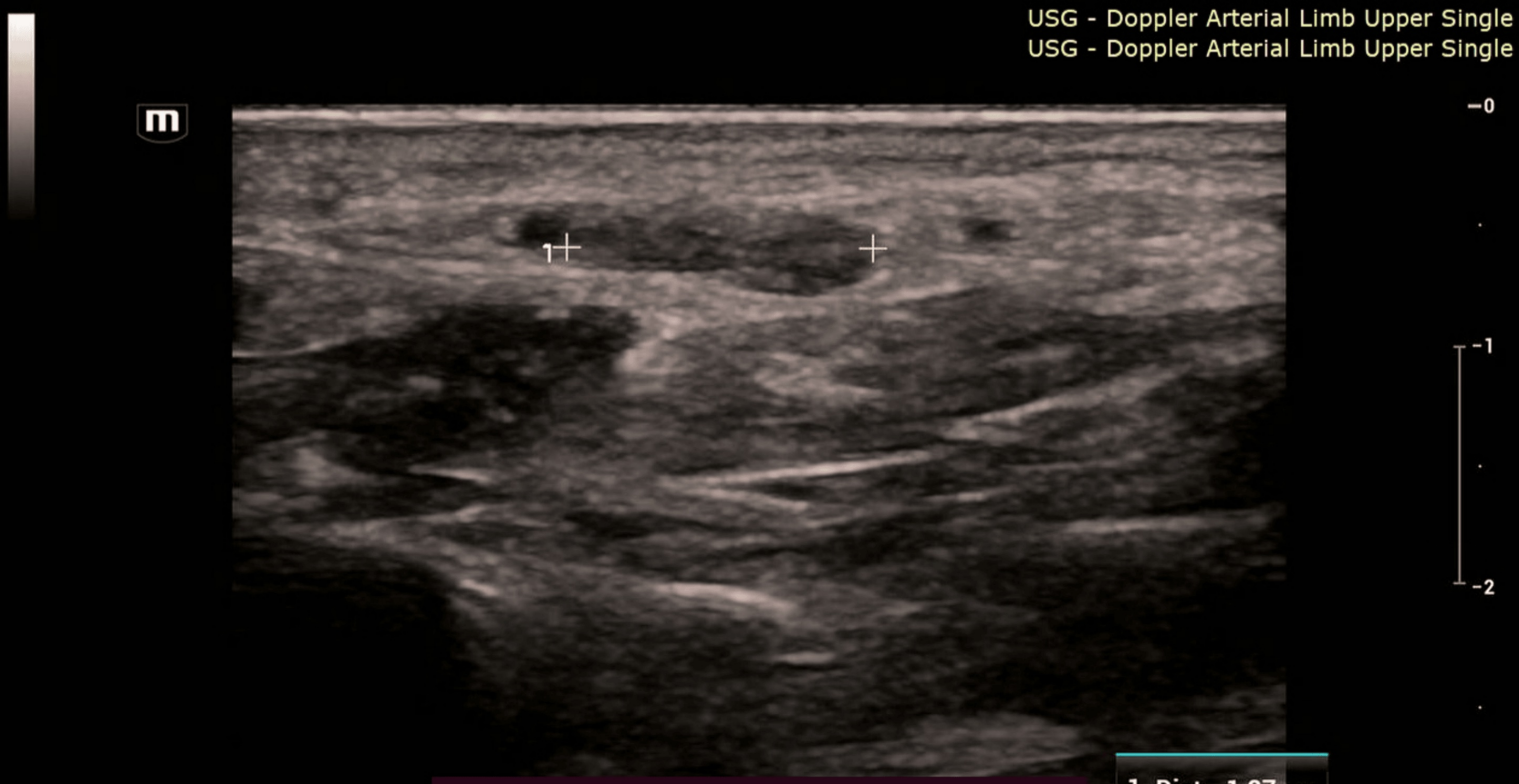
Arterial doppler of hand. Arterial Doppler revealed a short segment acute thrombus/embolus in the superficial palmar arch with absent distal flow signals, preserved proximal flow, and no significant proximal vessel stenosis.

A 31-year-old female, one month postpartum following a lower segment cesarean section, presented to the emergency department on 10 October 2023 with a fever of almost two weeks' duration and gradually worsening shortness of breath. She had experienced a high-grade fever with chills, which had made her increasingly weak and unwell. Upon inspection, she had small splinter hemorrhages on her fingernails, and palmar erythema was noted. On auscultation, she had an early diastolic murmur in the aortic region. Fever combined with a newly found murmur is highly suspected infective endocarditis [8]. To validate this suspicion, a trans-thoracic echocardiography on the same day was done, which revealed a vegetation of 1.3 × 0.6 cm on the aortic valve as shown in Figure 1. She was admitted to the cardiology department, and routine blood investigations and blood cultures were sent. Blood counts were normal, but her CRP and procalcitonin were found to be elevated. Blood cultures grew *Enterococcus faecalis*, common in genitourinary flora, confirming infective endocarditis [9]. She was started on culture-sensitive intravenous antibiotics and planned for elective aortic valve replacement after completing four weeks of therapy. On day eight of admission, she developed pain in the left hand. Arterial Doppler was performed, and it revealed a short-segment acute thrombus/embolus in the superficial palmar arch with absent distal flow signals, preserved proximal flow, and no significant proximal vessel stenosis, as shown in Figure 2. Since there was a good collateral supply and no tissue damage, it was managed conservatively [10].

**Figure 3:**
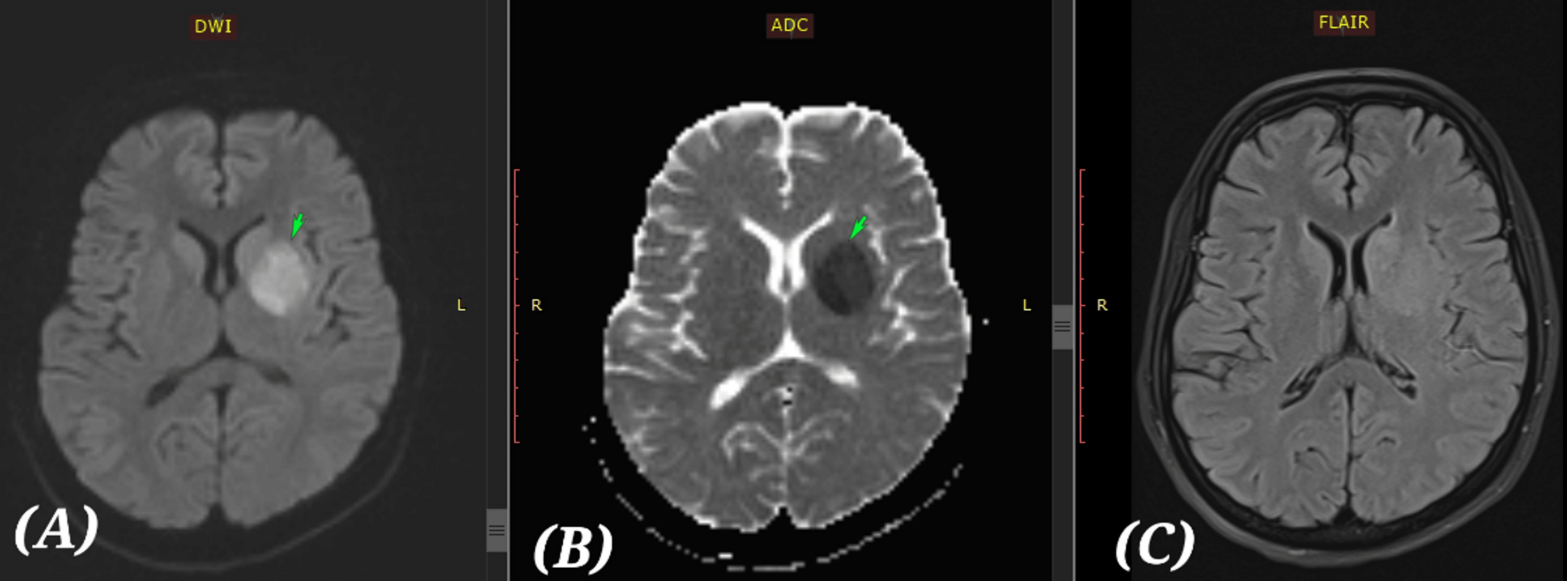
MRI brain-stroke protocol. Acute left MCA territory infarct, largest at the ganglio-capsular corona radiata region. (A) Diffusion-weighted imaging (DWI) showing a bright hyperintense signal (arrow marked) in the left basal ganglia/internal capsule region consistent with restricted diffusion suggestive of acute infarct. (B) Antibody-drug conjugate (ADC) with a corresponding dark hypo-intense area on the ADC map at the same site confirms true restricted diffusion suggestive of acute ischemic infarct. (C) Fluid-attenuated inversion recovery (FLAIR): There is no obvious bright signal seen in the same area, which means the infarct is very early (hyperacute, <4.5 hrs), before FLAIR changes appear.

**Figure 4:**
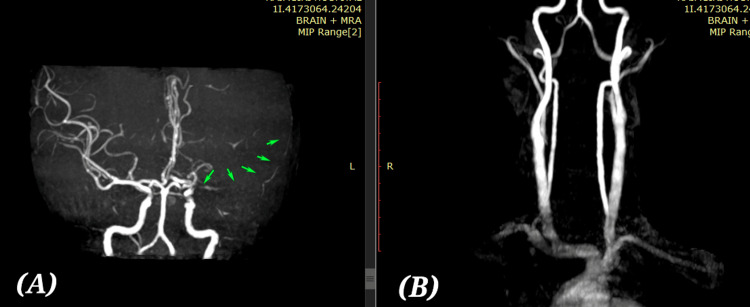
MR angiogram. Acute left MCA territory infarct, largest at the ganglio-capsular coronaradiator region. Severe near-total occlusion at the origin of the M1 segment with attenuated mid-segment signal and absent distal flow. No flow-limiting stenosis in other intra- or extracranial vessels (A) Intracranial MRA: Green arrows are pointing to abnormal attenuation of MCA, and its branches on the left side suggest stenosis/occlusion of proximal MCA. (B) Extracranial MRA: Bilateral carotid and vertebral arteries appear patent without obvious stenosis or occlusion. No flow-limiting lesions in the extracranial carotid system.

On 25 October 2023, the patient experienced a sudden onset of slurred speech and weakness in the right upper and lower limbs. At approximately 4:30 PM, clinical examination revealed right-sided hemiparesis, with muscle strength graded as 4/5 in the lower limb and 3/5 in the upper limb according to the Medical Research Council (MRC) scale. She also demonstrated mild dysarthria. Given the acute presentation, an MRI of the brain along with MR angiography using the stroke protocol was promptly performed to evaluate the underlying cause [11]. The MRI (Figures 3A-3C) and MR angiogram (Figures 4A, 4B) revealed an acute left middle cerebral artery (MCA) territory infarct, largest at the ganglio-capsular and corona radiata regions. Severe near-total occlusion at the origin of the M1 segment with attenuated mid-segment signal and absent distal flow. No flow-limiting stenosis in other intra- or extracranial vessels.

**Figure 5:**
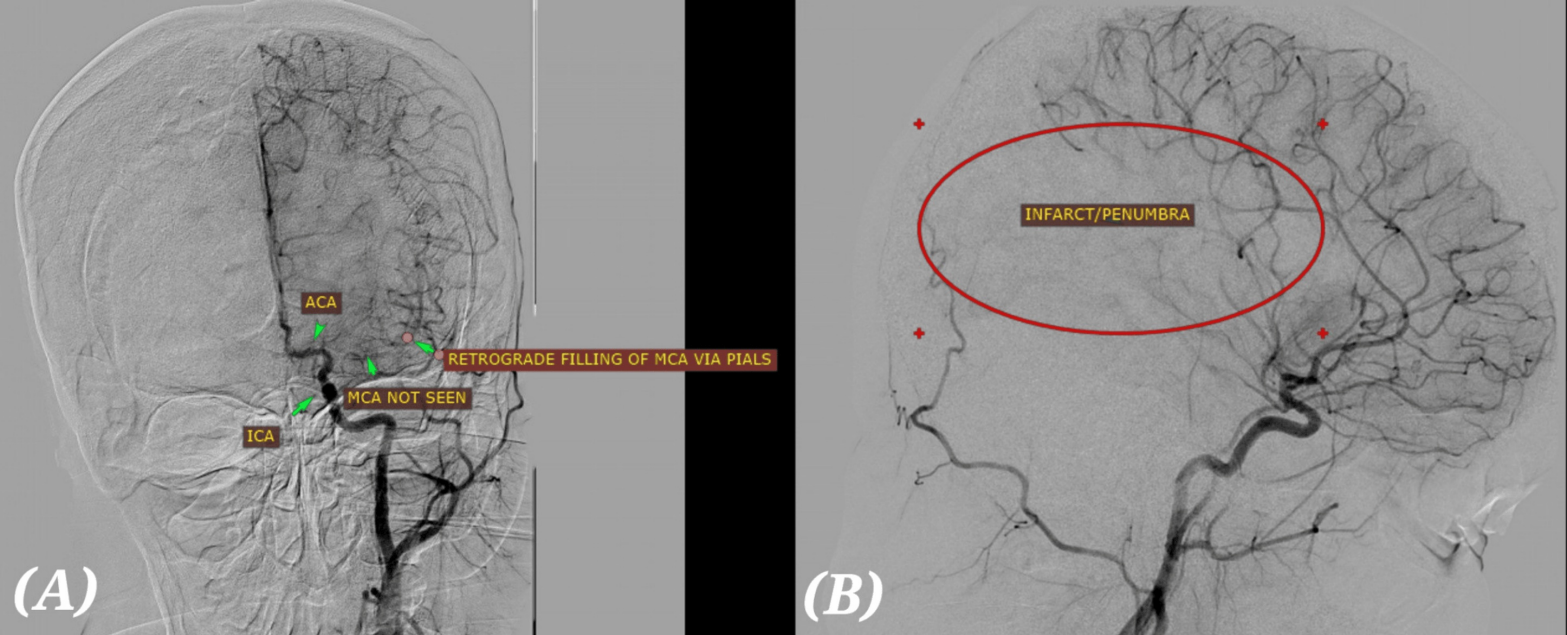
DSA before mechanical thrombectomy. (A) AP (anteroposterior) projection. (B) Lateral projection. The left middle cerebral artery (MCA) is not visualized in its expected course, suggesting MCA occlusion at the proximal M1 segment. Retrograde filling of MCA branches via pial collaterals from anterior cerebral artery (ACA) indicates collateral circulation is trying to perfuse MCA territory. An infarct/penumbra zone is seen in the MCA territory (highlighted in a red oval in the lateral view); this corresponds to ischemic brain tissue. DSA: Digital subtraction angiography

**Figure 6:**
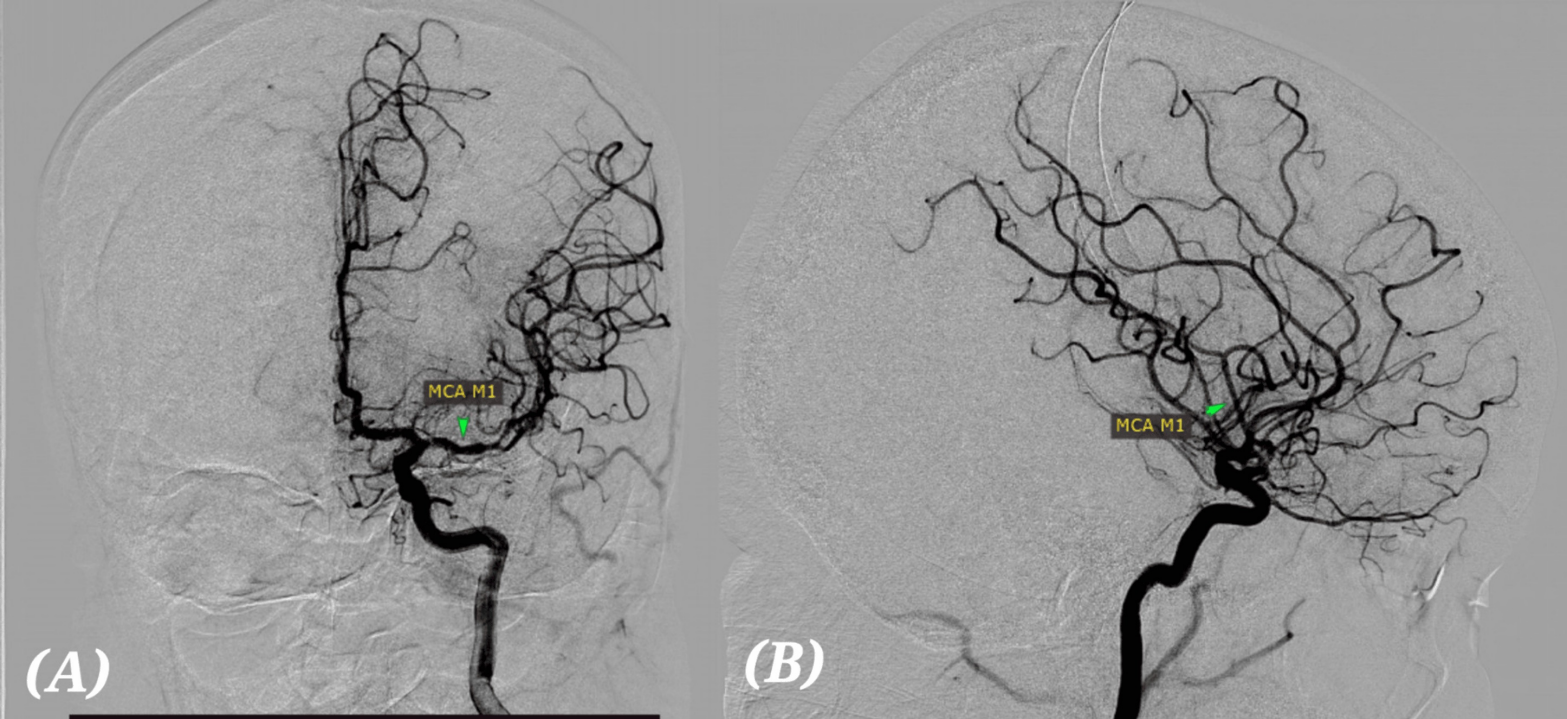
DSA after mechanical thrombectomy. (A) AP (anteroposterior) projection. (B) Lateral projection. The MCA M1 segment is now clearly seen (labeled), indicating that the previous occlusion in the MCA has been successfully recanalized. Normal antegrade flow into MCA branches is restored; the distal cortical branches of the MCA are now visible. Complete reperfusion (TICI grade 3) of MCA territory on both AP and lateral views. DSA: Digital subtraction angiography

In view of the acute large vessel occlusion, the patient was immediately taken up for emergency percutaneous mechanical thrombectomy at 5:00 PM. DSA confirmed complete M1 segment occlusion (Figures 5A, 5B). A single-pass recanalization was achieved using the SOLUMBRA technique, resulting in complete reperfusion (TICI grade 3) [12]. After mechanical thrombectomy, complete recanalization was achieved, as seen in the post-mechanical thrombectomy DSA (Figures 6A, 6B). Following the intervention, the patient showed a rapid and remarkable neurological recovery. Within the first 24 hours after the procedure, there was a notable improvement in speech fluency with a marked reduction in dysarthria. Motor function in the right upper and lower limbs steadily improved, with muscle strength increasing from an initial MRC grade of 3/5 in the upper limb and 4/5 in the lower limb immediately after the stroke to 4+/5 by the third postoperative day. Sensory deficits diminished significantly, and the facial asymmetry resolved almost completely. By the end of the first week, the patient had regained independent mobility, and no new neurological deficits or complications were observed during her hospital stay [13].

**Figure 7:**
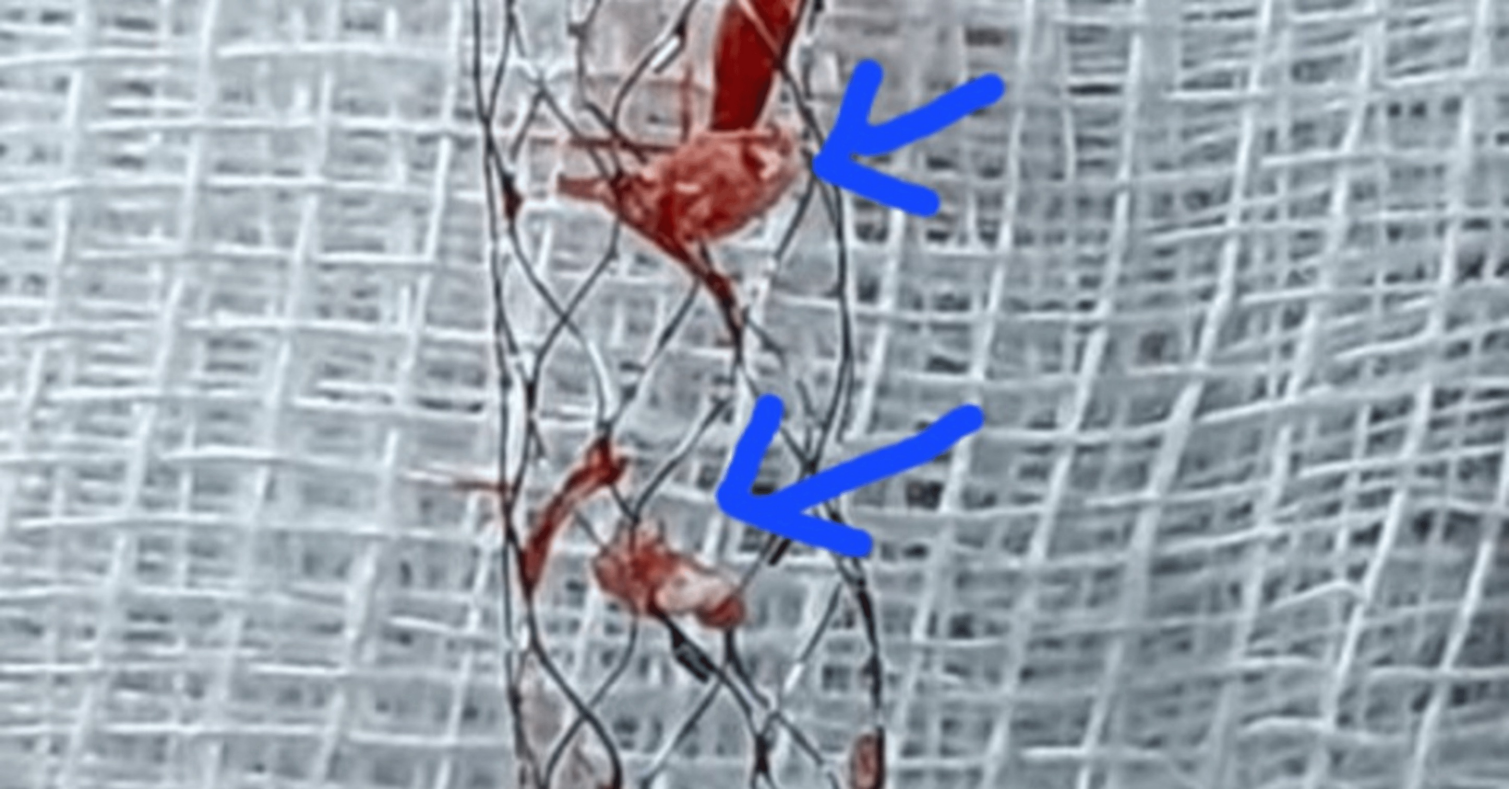
Mechanical thrombectomy specimen. Histopathology report Organized thrombus (fibrinous material with dense acute inflammatory cells composed of neutrophils, few lymphocytes, eosinophils, and focal calcification) with gram-positive *cocci* on staining.

The specimen retrieved during the mechanical thrombectomy (Figure 7) was submitted for histopathological examination and analysis. The histopathology report described an organized thrombus consisting of fibrinous material with dense acute inflammatory cell infiltration, predominantly neutrophils, along with a few lymphocytes, eosinophils, and areas of focal calcification. Staining further demonstrated the presence of gram-positive *cocci* [14].

In view of recurrent embolic episodes, an early aortic valve replacement was undertaken on 31 October 2023.

## Discussion

This case highlights the occurrence of multiple embolic events in the setting of infective endocarditis (IE), which in this patient included both peripheral arterial occlusion and ischemic stroke. *Enterococcus faecalis* is a pathogen in postpartum infective endocarditis, and its occurrence in this group may be associated with exposure of the genitourinary tract to bacterial colonization during the peripartum period. The presence of large vegetations, particularly those measuring more than 10 mm on the aortic valve, is known to markedly increase the risk of embolic events due to their greater likelihood of fragmenting and traveling to distant vascular territories [15].

A prompt diagnosis, achieved through the use of echocardiography in conjunction with positive blood cultures, plays a crucial role in guiding treatment decisions and reducing complications. Close clinical surveillance is necessary to detect embolic phenomena early, as these can develop at any stage of the disease. Multidisciplinary collaboration between cardiology, neurology, infectious disease, and cardiovascular surgery teams is essential for optimal outcomes.

In this case, mechanical thrombectomy demonstrated its value as a safe and effective option for treating acute large vessel occlusions secondary to IE, even though such interventions carry the theoretical risk of dislodging or disseminating infected embolic material [16]. Nevertheless, in carefully selected patients, the benefits of reperfusion outweigh these risks. Furthermore, early surgical intervention should be strongly considered, particularly in cases where embolic events recur despite appropriate antimicrobial therapy, as this strategy can help prevent further complications and improve long-term prognosis [17].

## Conclusions

This case illustrates the phenomenon of recurrent systemic embolic events in infective endocarditis (IE) involving both peripheral arterial occlusion and acute ischemic stroke. Both of these complications are due to the fragmentation of friable vegetations into the systemic circulation. In the patient presented here, the causative pathogen was found to be *Enterococcus faecalis*, a known pathogen of postpartum IE, potentially related to peripartum genitourinary exposure and transient bacteremia.

Extensive vegetations, especially those greater than 10 mm on the aortic valve, significantly raise embolic risk. It highlights the need for early and correct diagnosis by echocardiography and blood cultures, along with careful observation for recurrent embolization despite antibiotic treatment. Best management involves combined multidisciplinary care by cardiology, neurology, infectious disease, and cardiothoracic surgery. Mechanical thrombectomy, while conventionally taken cautiously in the setting of IE-related stroke, is safe and beneficial in selected large-vessel occlusive cases with rapid reperfusion and better neurological outcomes. Lastly, early valve surgery should also be undertaken in patients with ongoing embolization despite receiving antimicrobial treatment, as it treats the infection and risk of recurrence of embolic events and hence improves prognosis. Going forward, emphasis should be placed on early detection, close monitoring, and timely surgical repair in the first embolism event.

## References

[1] Cahill TJ, Prendergast BD (2016). Infective endocarditis. Lancet.

[2] Schuermann H, von Rennenberg R, Riegler C (2024). Characteristics associated with occurrence of stroke in patients with infective endocarditis - a retrospective cohort study. Neurol Res Pract.

[3] Vilacosta I, Graupner C, San Román JA (2002). Risk of embolization after institution of antibiotic therapy for infective endocarditis. J Am Coll Cardiol.

[4] Tzoumas A, Kousi T, Tsagalou EP (2024). Enterococcus faecalis as a cause of infective endocarditis - systematic review of case reports. Stu Med.

[5] Miller EC, Gatollari HJ, Too G (2016). Risk of pregnancy-associated stroke across age groups in New York State. JAMA Neurol.

[6] Muñoz P, Kestler M, De Alarcon A (2015). Current epidemiology and outcome of infective endocarditis: a multicenter, prospective, cohort study. Medicine (Baltimore).

[7] Kang DH, Kim YJ, Kim SH (2012). Early surgery versus conventional treatment for infective endocarditis. N Engl J Med.

[8] Jackson G, Camargo C, Ling LF (2013). The clinical picture: fever, dyspnea, and a new heart murmur. Cleve Clin J Med.

[9] Jahic M (2022). Aerobic vaginitis caused by Enterococcus Faecalis - clinical features and treatment. Mater Sociomed.

[10] Chitte SA, Veltri K, Thoma A (2003). Ischemia of the hand secondary to radial artery thrombosis: A report of three cases. Can J Plast Surg.

[11] Grecu N, Tiu C, Terecoasa E, Bajenaru O (2014). Endocarditis and stroke. Maedica (Bucur).

[12] Cho JS, Kim SU, Kim HJ (2019). Successful mechanical thrombectomy using solumbra technique in a 35-year-old man with achondroplasia: a case report. J Cerebrovasc Endovasc Neurosurg.

[13] Huimin Zhao, Qinrong Xu, Peng Chen Late neurological improvement during hospitalization is a predicative factor for acute ischemic stroke. Eur Jr Med Res.

[14] O'Connor BS KP, Perez MD GS, Ray MDR (2019). Histopathological examination of an embolus in infective endocarditis: Case report and review of the literature. Int Neu.

[15] Vilacosta I (2023). About vegetation size and its clinical implications. Cardiol J.

[16] Mowla A, Abdollahifard S, Sizdahkhani S (2022). Endovascular treatment of large vessel occlusion strokes caused by infective endocarditis: a systematic review, meta-analysis, and case presentation. Life (Basel).

[17] Ambrosioni J, Urra X, Hernández-Meneses Hernández-Meneses (2018). Mechanical thrombectomy for acute ischemic stroke secondary to infective endocarditis. Clin Infe Dis.

